# Dry computational approaches for wet medical problems

**DOI:** 10.1186/1479-5876-12-26

**Published:** 2014-01-25

**Authors:** Frank Emmert-Streib, Shu-Dong Zhang, Peter Hamilton

**Affiliations:** 1 Computational Biology and Machine Learning Laboratory, Center for Cancer Research and Cell Biology, School of Medicine, Dentistry and Biomedical Sciences, Queen’s University Belfast, Lisburn Road 97, Belfast, UK; 2 Center for Cancer Research and Cell Biology, School of Medicine, Dentistry and Biomedical Sciences, Queen’s University Belfast, Lisburn Road 97, Belfast, UK

**Keywords:** Computational genomics, Systems medicine, Genomics, Translational bioinformatics, Network medicine, Pharmacogenomics

## Abstract

This is a report on the 4th international conference in ‘Quantitative Biology and Bioinformatics in Modern Medicine’ held in Belfast (UK), 19–20 September 2013. The aim of the conference was to bring together leading experts from a variety of different areas that are key for Systems Medicine to exchange novel findings and promote interdisciplinary ideas and collaborations.

## Introduction

Breathtaking technological progress fueled by the human genome project [1] enables nowadays a quantitative, data-driven approach to study, not only basic biological processes, but also biomedical and clinical questions. For this reason, computational statistics approaches are needed for analyzing, integrating and interpreting high-throughput genomics data.

The 4th international conference in ‘Quantitative Biology and Bioinformatics in Modern Medicine’ aimed to foster quantitative research in Translational Bioinformatics comprising Computational Biology, Biomedical Informatics, Systems Biology and Network Medicine, with particular focus on disease-related problems and especially cancer. Due to the interdisciplinary character of the subject the invited speakers came from Medicine, Clinical Sciences, Biology and Computational Biology to provide a balanced overview of different aspects of the ‘same’ underlying problem. Also the audience had a diverse background making it truly a contemporary translational meeting.

On an organizational note, the registration fee for students was greatly reduced in order to encourage students to participate in the meeting. Given the still traditionally oriented curriculum of most university courses the meeting served also an educational mission by providing an object-lesson in interdisciplinary research.

## Genomic medicine enables medicine on the systems level

The welcome address of the conference was provided by the Dean of the School of Medicine, Dentistry and Biomedical Sciences, the newly elected President and Vice-Chancellor of the Queen’s University Belfast, Professor Patrick Johnston. Professor Johnston emphasized the general importance of computational & systems biology approaches to understand causal disease mechanisms on a genomic scale. Furthermore, he reminded at the beginnings of the conference that was initially funded by the Department for Employment and Learning (DEL, UK) to support the establishment of a Computational Biology infrastructure in Belfast.

It is important to emphasize that Genomic Medicine sets all boundary conditions that allows a systems analysis of medicine. However, many approaches still do not utilize available data up to their full potential but use them in a traditional, reductionist manner, e.g., by neglecting correlation structures among proteins or SNPs. Instead, the talks presented in this session showed original and creative approaches for Genomic Medicine on the systems level.

The first talk of the conference with the title ‘Modeling Endocrine Resistance in Breast Cancer’, which was also the keynote lecture, was presented by Robert Clarke (Biomedical Graduate Research Organization, Lombardi Comprehensive Cancer Center, Georgetown University Medical Center (USA)). The talk advocated the general perspective that a systems biology approach is required in order to integrate knowledge from cancer biology with computational and mathematical methods. As a specific case study, endocrine resistance in breast cancer was discussed adopting a network medicine approach [2]. A thought provoking conclusion from the presented analysis was that endocrine resistance may not require many new genes for its explanation, but just a few changes in the usage of existing interactions among known genes.

Francesca Ciccarelli (Department of Experimental Oncology, European Institute of Oncology, IFOM-IEO Campus (Italy)) presented a talk about ‘Genomics and Network Biology to Identify Systems level properties of cancer genes’. In her talk common mutations in cancer genes were discussed with the goal to identify *driver genes* and novel therapeutic targets. As a result it was found that cancer genes form interconnected hubs in the human protein-protein interaction (PPI) network and are broadly expressed, in particular in the cancer tissue where they mutate. Furthermore, recessive cancer genes are old on an evolutionary scale and form singleton hubs, whereas dominant cancer genes are fairly recent present in the PPI network as duplicated hubs [3].

Christos Hatzis (Yale Comprehensive Cancer Center, Yale School of Medicine (USA)) gave a talk about ‘Complexity and limits of predictability in breast cancer’. Christos’ presentation started by emphasizing different types of heterogeneity, e.g., inter-tumor and intra-tumor heterogeneity, in breast cancer and methods for their characterization. In the following, different breast cancer subtypes were studied quantitatively and basal-like tumors were found to be more heterogeneous than HER2 or Luminal A and B. On a general note, it was suggested to view the heterogeneity of tumors more as a feature rather than a nuisance in order to exploit this information.

## Integrating data of different molecular and clinical data sets

The important problem of data integration was addressed by Sampsa Hautaniemi (Centre of Excellence in Cancer Genetics, Faculty of Medicine, University of Helsinki (Finland)) who gave a presentation with the title ‘Analysis and integration of large-scale molecular and clinical data in cancers’. Sampsa showed a study that integrated transcriptional and clinical data of high-grade serous ovarian cancer patients to identify molecular cause for platinum resistance, which forms the standard chemotherapy. As a result from this integrative analysis TR3 and its connection to signaling pathways were identified.

Also Andy Sims (Applied Bioinformatics of Cancer, University of Edinburgh (UK)) presented a talk about the integration of data with the title ‘Gene expression data integration for breast cancer research’. However, in this talk the integration of different data sets of the same type were the central theme [4]. Specifically, the integration of gene expression data from breast cancer were discussed with a particular consideration of *batch effects* and the reproducibility of results.

The following talk was a student presentation by Jaine Blayney (Centre for Cancer Research and Cell Biology, Queen’s University, Belfast, UK - now Lecturer at University of Ulster (UK)) taking about the ‘Determination of the authenticity and lineage of cell lines using compositional gene expression profiles’.

## Network as a biomarker of the system

The first contribution discussing modern biomarker approaches was given by Richard Kennedy (Center for Cancer Research and Cell Biology, Queen’s University Belfast (UK)) who gave a talk with the title ‘Discovery and Validation of a Predictive Biomarker for Breast Cancer Chemotherapy’. The talk reported the identification of a 44 gene signature biomarker corresponding to DNA damage response deficiency (DDRD). Statistical as well as experimental validations were presented and the biological basis of the DDRD 44 biomarker were investigated leading to a connection with loss of the FA/BRCA pathway [5,6].

Sol Efroni (The Mina and Everard Faculty of Life Science, Bar Ilan University (Israel)) followed with a talk entitled ‘The Network is a Biomarker in Cancer Signatures’. Sol advocated the interesting hypothesis that the networks underlying pathways could be used as biomarkers. By studying gene expression data from glioblastoma multiforme (GBM) and ovarian cancer the p38/MAPKAP pathway and the PDGF signaling pathway were reported to lead to a robust prognostic stratification [7,8].

The next talk was again a student presentation given by Fabio Liberante (Centre for Cancer Research and Cell Biology, Queen’s University, Belfast, UK) with the title ‘Identification of novel therapies in the treatment of MDS/AML using a signature of disease development and progression with the sscMap tool’. He reported his recent work on the creation of a gene signature representing the progression of disease state from healthy condition to MDS then AML. Using the sscMap (Statistically Significant Connections’ Map) tool [9], a number of compounds were identified as having the potential to reverse the disease state progression. His talk showcased a success of applying a Quantitative Biology and Bioinformatics approach to the identification of novel use for an existing drug.

## Pharmacogenomics and drug identification

Ann Daly (Institute of Cellular Medicine, Newcastle University Medical School (UK)) gave the first talk in the Pharmacogenomics and drug identification session, with the title ‘Use of GWAS and exome sequencing to identify genes relevant to drug-induced liver injury’. After a background introduction of idiosyncratic adverse drug reactions, she reported on the UK-wide study on drug-induced liver injury used Drug-induced liver injury (DILI), for which she serves as a coordinator. The aim of the study was to find genes predisposing to DILI using GWAS (genome wide association study) and exome sequencing. Highly significant associations with particular HLA genotypes were reported for idiosyncratic drug-induced liver injury with several specific drugs, and the possible underlying mechanisms were discussed. Her talk represented interesting showcases where Genomics has played an important role in increasing our understanding of individual risk for idiosyncratic drug-induced liver injury.

Munir Pirmohamed (Department of Molecular and Clinical Pharmacology, The Wolfson Centre for Personalised Medicine, Institute of Translational Medicine, University of Liverpool (UK)) followed on the theme on adverse drug reactions by giving a talk with the title ‘Genomics of adverse drug reactions: the need for a multi-functional approach’. He argued for the need to develop multi-functional approaches to find solutions to prevent Adverse Drug Reactions (ADRs), and described the efforts of the international Serious Adverse Event Consortium (iSAEC) in developing phenotype standardization for a number of phenotypes in order to facilitate the translation of genomic predictors into clinical practice. He also discussed the issue of different evidence standards that are currently applied to non-genetic versus genetic tests. Using examples of drug exposure and drug adverse reaction, he highlighted the substantial differences between the level of evidence required for pharmacogenetic tests and that for non-genetic diagnostic tests. As these are among the challenges holding back the translation of systems medicine, Munir Pirmohamed’s talk helped to raise awareness of some existing bottleneck. He argued that a level playing field for pharmacogenetic tests could help accelerate the delivery of a more personalized medicine.

Through a video link, Aravind Subramanian (Broad Institute of Harvard and MIT (USA)) gave a talk on the LINCS (Library of Integrated Network-based Cellular Signatures) project, which aims to create a network-based understanding of biology by their systematic efforts to generate over 1M gene expression profiles using chemical or genetic perturbagens. Aravind described the development of L1000 assay that has dramatically reduced the cost of gene expression profiling. It was reported that the current LINCS dataset contains over 1.2M gene expression profiles, covering over 5k small molecule and 3k genetic perturbagens. Data access was through multiple levels of data matrices and cloud-compute beta has been released. The utility of the LINCS dataset was demonstrated with several examples of emerging scientific findings.

Benjamin Haibe-Kains (Bioinformatics and Computational Genomics Laboratory, Institut de Recherches Cliniques de Montreal (Canada)), in his stimulating talk ‘Are pharmacogenomic studies useful for developing genomic predictors of drug response?’, reported his recent work on comparing the datasets from several large-scale pharmacogenomics studies [10]. The high-throughput genomic data were well correlated, but he reported that the measured pharmacological response to drug is poorly correlated among these different studies. He suggested that it was difficult to draw firm conclusions from the discordant pharmacological responses in the different studies. Furthermore, the validity and implications of using IC50 and AUC as outcome measures to assess gene-drug relationships was discussed. This study raised important concerns and it remains to be seen how these problems can be overcome.

## Poster awards

The conference finished with awards for the best poster presentations that were voted by the participants of the conference. The first prize went to Fabio Liberante (Center for Cancer Research and Cell Biology, Queen’s University Belfast (UK)) with a poster on the same topic that was selected for his oral presentation (see above). The second prize went to Qing Wen (Center for Cancer Research and Cell Biology, Queen’s University Belfast (UK)) with a poster on ‘Gene signatures for connectivity mapping to target disease phenotypes’, which describes the development of a standardized procedure and protocol for creating disease gene signatures and their applications to cancer research. Both students received a book voucher that was generously sponsored by Cambridge University Press.

## Conclusions

Due to the limited time frame of our conference the contributed talks could not touch upon all aspects that are of importance for Translational Medicine. For example, Medical Bioinformatics [11] approaches for electronic patient record data or Computational Physiology [12] models for various biological systems, including drug response, are currently of great importance. Nevertheless, an important message of the meeting that goes beyond the individually contributed talks is that Genomic Medicine does not only rely on *computational approaches* for the number crunching of the data, but also as an intellectual driving force. The latter relates, e.g., to the experimental design of a study for ensuring the generated genomics data are actually usable for a systems approach rather than representing an accumulation of massive amounts of reductionist data. Another example is the usage of networks as a biomarker [13] and the demonstration that such an abstract mathematical object as a network can actually have a very practical medical application.

Furthermore, the conference had a social agenda bringing together experts from the dry and wet labs in a small meeting, without parallel sessions, forcing them to talk and interact with each other. Despite the general acknowledgement that Genomic Medicine is an interdisciplinary endeavor the communication between scientists from these different worlds still needs improvement because without a genuine understanding and appreciation of each other the patients will not get the best possible treatment that could be achieved collaboratively.

If the future of biology is dry in the sense of [14] remains to be seen. However, it is unquestionable that computational approaches in Biomedicine are gaining more and more importance in the near future, particularly in Translational Bioinformatics.

## Competing interests

The authors declare that they have no competing interests.

## Authors’ contributions

FES, SDZ and PH wrote the text and approved the final version.
